# Urgent psychiatric consultations for suicide attempt and suicidal ideation before and after the COVID-19 pandemic in an Italian pediatric emergency setting

**DOI:** 10.3389/fpsyt.2023.1135218

**Published:** 2023-06-30

**Authors:** Massimo Apicella, Giulia Serra, Monia Trasolini, Elisa Andracchio, Fabrizia Chieppa, Roberto Averna, Maria Elena Iannoni, Antonio Infranzi, Marianna Moro, Clotilde Guidetti, Gino Maglio, Umberto Raucci, Antonino Reale, Stefano Vicari

**Affiliations:** ^1^Child Neuropsychiatry Unit, Department of Neuroscience, Bambino Gesù Children's Hospital, Istituto di Ricovero e Cura a Carattere Scientifico (IRCCS), Rome, Italy; ^2^Department of Emergency, Acceptance and General Pediatrics, Bambino Gesù Children's Hospital, Istituto di Ricovero e Cura a Carattere Scientifico (IRCCS), Rome, Italy; ^3^Department of Life Sciences and Public Health, Catholic University, Rome, Italy

**Keywords:** COVID, adolescent, mental health, suicide, attempt, depression, emergency care

## Abstract

**Introduction:**

Suicidal attempts (SAs) in youth have been increasing during the last decades.

**Methods:**

We studied consultations, SA, and suicidal ideation (SI) in a pediatric emergency department (ED).

**Results:**

From 1 January 2011 to 31 May 2022, 606,159 patients accessed the ED, 8,397 of who had a child psychiatry consultation (CPC). CPCs increased significantly by 11 times in the last decade (155 in 2011 vs. 1,824 in 2021, *p* < 0.001); CPCs for SA increased significantly by 33 times, from 6 in 2011 to 200 in 2021 (3.9% of total CPC vs. 11%, *p* < 0.001). While total CPCs increased constantly during the entire period (annual percent change (APC) of 21.7 from 2011 to 2021 in a 0 joinpoint model), CPCs for SA increased significantly from 2011 to 2016, were approximately stable from 2016 to 2020, and then had a peak in 2021 after the COVID-19 pandemic (APC from 2011 to 2016 of 64.1, APC of 1.2 from 2016 to 2020, and APC of 230 after 2020 in a 2-joinpoint model).

**Discussion:**

Total CPCs in ED as well as evaluation for SA and SI increased significantly during the last decade. CPCs for SA had an additional increase after the COVID-19 pandemic. This picture warrants timely and efficient improvements in emergency settings and mental health resources.

## 1. Introduction

Suicide is a multidimensional phenomenon resulting from the interaction between biological, genetic, psychological, and environmental factors (1). Suicidal thoughts and behaviors are relatively rare in childhood, but they increase significantly during the transition to adolescence and are a major public health concern for populations of this age group (2). Many factors have been called into question to explain the current prevalence of suicidal behaviors in adolescents, beginning from the apparent increased incidence of specific psychiatric disorders in this age group. Depression, anxiety, and substance use disorders, as well as non-suicidal self-injury (NSSI), have all been significantly correlated with suicidal behaviors and have become more prevalent in adolescents in the last decades (3). Solid evidence has been accumulated on impulsiveness and difficulties in interpersonal and intrapersonal emotional regulation as the most remarkable dimensions underlying adolescent SA and NSSI (4–6).

A variety of psychosocial risk factors have been associated with suicidal behaviors in both children and adolescents. These include a family history of suicide, poor family relationships, and restricted educational achievements (7). Among the most widely studied psychosocial risk factors, school failure, low self-esteem, poor peer relationships, poor family relationships, sexual abuse, and intrafamilial violence have a confirmed prominent effect on suicidal risk (8). Previous suicidal attempts (SAs) and NSSI are considered two of the strongest risk factors for suicide in most studies, as well as in longitudinal cohorts (9–11).

Over the past decade, an increase in mental health problems has been observed in children and adolescents in developed countries. Data from emergency care settings revealed a rising trend for anxiety and mood disorders (12). Epidemiological studies from high-income countries reveal an increasing trend for affective disorders in youth since the last decade, which is more marked for female adolescents (13). Studies from hospital settings also revealed an increase in self-harm as a presenting reason for emergency care seeking between 2011 and 2014. Its occurrence is higher among girls aged 13–16 years (14). Moreover, suicide rates increased in the adolescent population between 15 and 19 years in England by 7.9% during the years between 2010 and 2017 (15). An increasing trend of suicides is also evident in the last 20-year interval in the United States (16).

In Italy, the overall incidence rate for suicide is estimated as 6.5/100,000 in 2015, which is lower compared to the European mean rates. For people under 24 years, the rate is 1.4/100,000 (17). Incidence is higher in men, in agreement with previous international literature which describes a higher incidence of SA in women and a higher incidence of completed attempts in men (2, 18). A previous study from our group reported an increased rate of emergency care seeking for SI, SA, and NSSI. Moreover, we already reported a growing demand for consultation on suicidal ideation, suicide attempts, and NSSI. The number of consultations for SA, SI, and NSSI among all psychiatric consultations switched from 12 (7.7% of total child psychiatry consultations, CPCs) in 2011 to 117 (19% of total CPCs) in 2016, and the increase was observed mainly among female adolescents and related to the diagnosis of major mood disorders (19).

The recent coronavirus disease 2019 (COVID-19) pandemic has had significant effects on mental health. These effects seem to be more marked on youngsters, and the consequences are supposed to persist for years (20). Moreover, for previous epidemics caused by severe acute respiratory syndrome, Ebola, and Middle East respiratory syndrome, adverse psychological consequences have been reported for adults and children (21).

Right after the pandemic outbreak, access to emergency care for any medical reason and for psychiatric care decreased in high-income countries (22). Possible explanations for the reduction of any medical consultations at the emergency departments include fear of infection with the new coronavirus. The reduction of psychiatric urgencies might be related to a decrease in scholastic requirements and continuous close parental supervision and sometimes is associated with the implementation of alternative ways to manage acute psychopathological events in some Italian regions (23). This reduction probably also represented a limit to help seeking from vulnerable youths during the first phase of the pandemic. Indeed, in the second pandemic period later in 2020 and in the first months of 2021, several authors reported an increase in psychiatric emergencies (24–29). Possible reasons for this phenomenon include economic uncertainty of parents, increased screen time (26), social isolation, bereavement, domestic violence, an increase in internalizing symptoms during lockdown (27, 30), as well as post-infective sequelae in some cases (30).

We performed the present study to investigate the effect of the recent COVID-19 pandemic on children and adolescents' mental health, analyzing the change in the trend of CPC after the pandemic and comparing it with the previous increasing trend. We focused on SA and SI in relation to a preexisting increasing trend before 2020. We collected data from one of the largest Italian pediatric hospitals until 31 May 2022 to have a broader view of trends of psychiatric urgencies and included the period of gradual resumption of all community activities after the end of the lockdown.

## 2. Materials and methods

### 2.1. Population and setting

We performed a retrospective chart review on access to the emergency department (ED) of Bambino Gesù Children's Hospital (Ospedale Pediatrico Bambino Gesù IRCCS, OPBG) in Rome from 1 January 2011 to 31 May 2022. OPBG is the largest pediatric hospital in Italy, and the ED admits patients coming both from Rome and the surrounding areas and from other cities and regions of southern Italy. Access to the ED is free and open 24/24 h for 7/7 days. For our analysis, we included data on patients (a) aged 0–18 years and (b) requiring a CPC in ED for any reason. For subjects with repeated evaluations during the period analyzed, data from repeated access were included.

Even if some municipalities in Italy were locked down at the end of February 2020, the region where our hospital is located was locked down at the beginning of March 2020 with the rest of the country (31). This is the reason why for our analysis we considered the months from March 2020 to May 2022 as the period after the start of the pandemic and the period from 2011 to February 2020 as the previous reference pre-pandemic period.

### 2.2. Measures

Subjects entering the ED for a CPC are systematically assessed both clinically and by a rating scale for suicidal ideation and behaviors. The Columbia suicide severity scale (C-SSRS) screening version is a screening instrument, consisting of a six-item clinically administered interview, which is routinely administered to all subjects in our ED during the CPCs. The C-SSRS is one of the most used instruments for the assessment of SI and suicidal behaviors. It is used in both clinical and research settings, in adolescents as in adults. It provides definitions of SI and SA that are widely accepted and shared by the current research and effectively distinguishes SA from NSSI (32). A subset of item measures inquires about suicidal ideation in the previous month and includes questions on 1) wish to be dead, 2) non-specific active suicidal thoughts, 3) suicidal thoughts with methods, 4) suicidal intent, and 5) suicidal intent with a plan. A second subset inquires about suicidal behaviors in the previous 3 months, and actual SAs, aborted or interrupted attempts are coded nominally. The timing of the suicidal behavior is recorded. Its use in ED settings is considered feasible and information derived from it such as the presence of suicidal ideation with a method (first three questions) or the presence of suicidal behavior inquired in the second subset has been demonstrated to correlate with increased odds of death by suicide (33). Suicidal behavior has been classified according to Posner (34). An attempt has been defined as any self-harming behavior resulting in any damage with non-zero intent to die, declared by the patient or evident from documented circumstances. An interrupted attempt has been defined as a behavior inevitably leading to a suicide attempt, interrupted by a person or external circumstance before resulting in any damage. Self-interrupted/aborted attempt is defined as a behavior inevitably leading to a suicide attempt, interrupted by the individual autonomously before resulting in any damage. Preparatory behavior is defined as any act prepared for the imminent performance of a suicide attempt, including access to a specific method and preparation to the perspective of own personal death. For the purposes of the present study, we considered patients with an ED evaluation for SA as a patient with at least one attempt or interrupted attempt during the 7 days preceding the CPC in ED. We considered a patient with an ED evaluation for SI as a patient with suicidal thoughts with a method, associated with some degree of intent to die and/or with a specific suicidal plan in the last month before access, according to the definitions of C-SSRS screening version, and without suicidal attempts during the 7 days preceding the evaluation.

### 2.3. Statistical analysis

Categorical variables have been presented as the rate and number of observations. Continuous variables have been tested using the Shapiro–Wilk test and, since not normally distributed, a non-parametric approach has been preferred. Continuous variables have been presented as median, minimum, and maximum values and interquartile range. To determine statistical differences between incidence rates in different years, the chi-square test was used for categorical variables. For continuous variables, the Mann–Whitney test or the Kruskal–Wallis test has been used, as appropriate. The analyses have been performed using Microsoft Excel and IMB SPSS v20.0.

Changes in the total number of annual evaluations and annual evaluations for SI and for SA from 2011 to 2021 were analyzed using a joinpoint regression model, based on a Poisson regression model for counts. The optimal number of joinpoints was selected using a permutation test (35). The slopes for each segment were converted to annual percentage changes, and their combined average, weighted for each segment, has been presented as annual percent change (APC). These analyses were performed using the Joinpoint Regression Program version 4.9.1.0 (36).

## 3. Results

### 3.1. Child psychiatry consultations for suicide attempt and suicidal ideation from 2011 to 2022 using univariate analysis

During the study period, 606,159 (on average 50,513 ± 9,897 per year) urgent medical evaluations, requested for any reason by parents or caregivers of children and adolescents aged from 0 to 18 years old, were performed. From January 2011 to May 2022, 8,397 CPCs were performed (on average 692 ± 478 per year), requested for psychiatric reasons by parents or caregivers of children and adolescents younger than 18 years (Table 1). The number of CPCs increased significantly by 11 times, ranging from 155 in 2011 to 1,824 in 2021, corresponding, respectively, to 0.33% of the total ED access in 2011 vs. 4.15% of the total annual ED access in 2021 (*p* < 0.001, Table 1).

**Table 1:** Number of urgent evaluations at the emergency department (ED) of OPBG from 2011 to 2022.

**Year**	**2011**	**2012**	**2013**	**2014**	**2015**	**2016**	**2017**	**2018**	**2019**	**2020**	**2021**	2022^*^	Mean (SD)^**^	**Total 2011–2022**	***p-value* (χ2 test)**
Total (N)	47,496	52,916	53,015	56,061	56,546	56,302	57,321	56,371	58,609	43,945	43,963	23,614	50,513 (SD 9,897)	606,159	N/A
CPC (*N, %* of Total)	155 (0.33%)	239 (0.45%)	237 (0.45%)	451 (0.80%)	541 (0.96%)	614 (1.1%)	715 (1.3%)	851 (1.5%)	1,059 (1.8%)	927 (2.1%)	1,824 (4.2%)	784 (3.3%)	692 (SD 478)	8,397 (1.4%)	*P < *0.001 (4,684)
CPC for SA (*N, %* of CPC)	6 (3.9%)	6 (2.5%)	12 (1.9%)	25 (5.5%)	34 (6.3%)	53 (8.6%)	56 (7.8%)	72 (8.5%)	52 (4.9%)	60 (6.5%)	200 (11%)	59 (7.5%)	52.4 (SD 54.1)	635 (7.6%)	p < 0.001 (62.3)
CPC for SI (*N, %* of CPC)	4 (2.6%)	1 (0.4%)	5 (2.1%)	14 (3.1%)	38 (7.0%)	45 (7.3%)	94 (13%)	68 (8.0%)	85 (8.0%)	124 (13%)	127 (7.0%)	82 (11%)	55.0 (SD 47.6)	687 (8.2%)	p < 0.001 (120)

Total, number of urgent evaluations in the ED by year; CPC, child psychiatric consultations, number of urgent psychiatric evaluations in the ED by year and percentage of the total urgent evaluations by year; CPC for SA, number of urgent psychiatric evaluations in the ED requested for a suicide attempt and percentage of the total psychiatric evaluations by year; CPC for SI, number of urgent psychiatric evaluations in the ED requested for a suicidal ideation and percentage of the total psychiatric evaluations by year.

^*^Data for the year 2022 are available from 1 January to 31 May.

^**^Mean (SD) are calculated from January 2011 to December 2021.

ED, emergency department; SA, suicidal attempt; SI, suicidal ideation.

From 1 January 2011 to 31 May 2022, at the emergency department of the OPBG, 7.6% (635/8,397; on average 52.4±54.1 per year) of the total CPCs were requested for children or adolescents referred to the ED for SA at least one time in the last 7 days. The number of CPCs for SA significantly increased by 33 times in the last decade, from six visits in 2011 to 200 in 2021 (3.9% vs. 11% of total CPC, respectively; χ^2^ = 62.3, *p* < 0.001) (Table 1).

From 1 January 2011 to 31 May 2022, at the emergency department of the OPBG, 8.2% (687/8,397; on average 55.0 ± 47.6 by year) of the total CPCs were requested for children or adolescents referred to the ED for SI. The number of CPCs for SI significantly increased by 32 times in the last 10 years, from four visits in 2011 to 127 in 2021 (2.6% vs. 7.0% of total CPC; χ^2^ = 120, *p* < 0.001) (Table 1).

### 3.2. Child psychiatry consultations for suicide attempt and suicidal ideation before vs. during the COVID-19 pandemic

The total number of CPCs requested at the emergency department of OPBG increased significantly from the pre-pandemic period to the pandemic period. The median number of CPCs increased from 45/month (range 13 to 119; interquartile range 20–60) in the pre-pandemic period to 136/month (range 51 to 198; interquartile range 79 to 156, U = 2,802.5, *p* < 0.001) in the pandemic period.

The median number of CPCs for SA in the pre-pandemic period was of 3.0/month (range 0 to 11; interquartile range 1.0–4.7) and increased significantly to 11/month (range 1 to 28; interquartile range 5–15, U = 2,633.0, *p* < 0.001) in the pandemic period.

The median number of CPCs for SI in the pre-pandemic period was of 3.0/month (range 0–13.0 interquartile range 0–6.0) and increased significantly to 11/month (range 3–20; interquartile range 9–14, U = 2,792.5, *p* < 0.001) in the pandemic period.

### 3.3. Joinpoint regression analysis on the total number of consultations, consultations for suicidal ideation, and consultations for suicidal attempts

**Figure 1:**
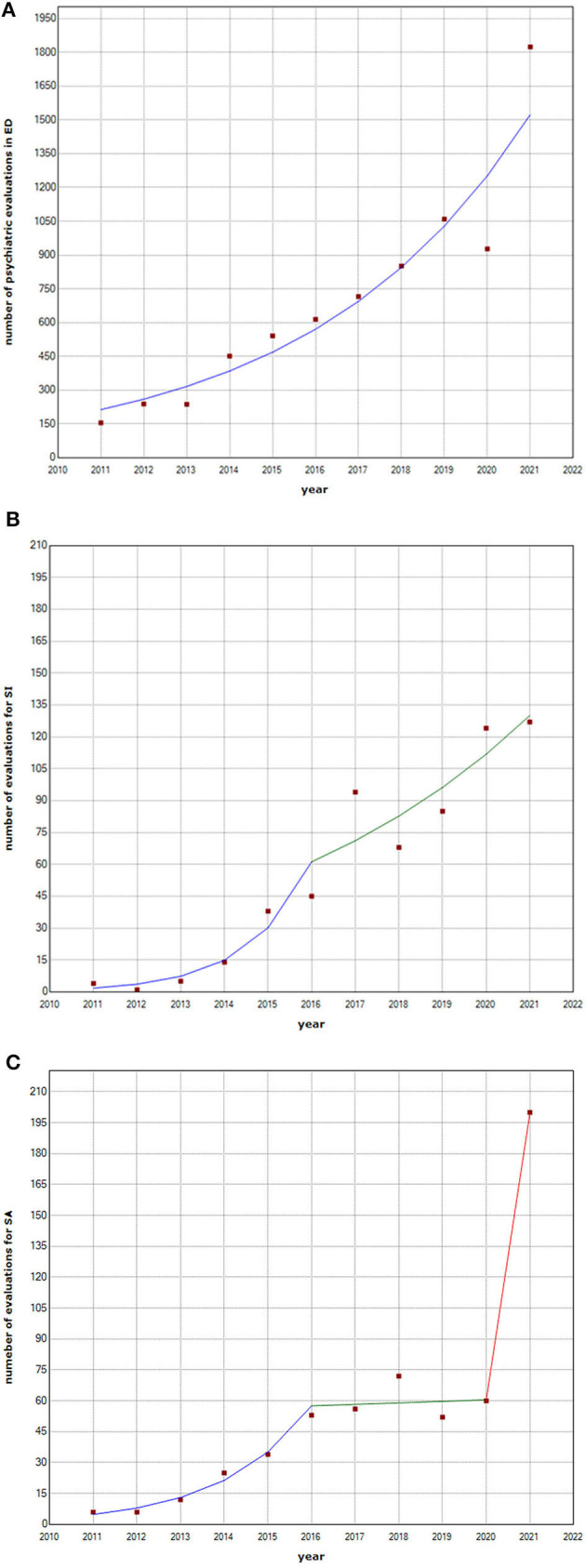
Total number of psychiatric evaluations in ED, number of evaluations for SI, and number of evaluations for SA per year. Joinpoint regression analysis, years 2011-2021. **(A)** Total number of psychiatric evaluations in ED per year: Joinpoint regression analysis, years 2011-2021. **(B)** Number of evaluations for SI per year: Joinpoint regression analysis, years 2011-2021. **(C)** Number of evaluations for SA per year: Joinpoint regression analysis, years 2011-2021. ED, emergency department; SA, suicidal attempt; SI, suicidal ideation.

The number of psychiatric evaluations in ED increased significatively and stably during the study period, without inflection points in the annual increase trend, starting from 155 in 2011 and reaching a number of 1,824 in 2021. The joinpoint regression analysis for this trend identified no joinpoints (*p*-value of the permutation tests >0.165) and an APC of 21.7 (95% CI = 18.2 to 25.4, t = 15.0, *p* < 0.001) for the entire time of the study from 2011 to 2021 (Figure 1A). The number of total annual pediatric evaluations in ED for any reason slightly decreased in the last years after a previous stably increasing trend. The number of total yearly evaluations, indeed, approximated a one-joinpoint model with a joinpoint in 2018 (*p*-value of the permutation tests < 0.001), with an APC of 2.3 (95% CI: 1.0 to 3.6, t = 4.4 p = 0.005), during the years from 2011 to 2018 and a following APC of −10.7 (95% CI: −16.3 to −4.7, t = −4.3 *p* = 0.005) from 2018 to 2021.

Evaluations for SI increased too, starting from 4 in 2011 to 127 in 2021, and their annual increase trend was significantly greater before 2016, followed by a subsequent slighter but constant increase. For this variable, indeed, the permutation test on joinpoint regression analysis identified a one-joinpoint model (*p*-value of the permutation tests was < 0.006), with a joinpoint in 2016. The APCs indicate that between 2011 and 2016, the SI evaluation rate increased with an APC of 102.8 (95% CI: 34.6 to 205.6, *t* = 4.2 *p* = 0.006), and during years from 2016 to 2021, it continued to increase with an APC of 16.3 (95% CI: 4.6 to 29.3, *t* = 3,5 *p* = 0.013) (Figure 1B).

Evaluations for SA, finally, also increased significantly during the study period, from 6 in 2011 to 200 in 2021, with a more complex trend. SA increased constantly from 2011 (6 SA) to 2016 (53 SA) and remained approximately stable until 2020 (60 SA). The following peak of 200 evaluations for SA in 2021 is significantly misaligned with the previous trend of 5-year stability. For evaluations for SA, indeed, the permutation test on joinpoint regression analysis identified a two joinpoints model (*p*-value of the permutation tests was < 0.001), with a joinpoint in 2016 and a joinpoint in 2020. The APCs indicate that between 2011 and 2016, the rate of evaluations for suicide attempts/total CPC increased with an APC of 64.1 (95% CI: 34.8 to 99.8, *t* = 7.0 *p* = 0.002); during years from 2016 to 2020, substantial stability with an APC of 1.2 (95% CI: −19.6 to 27.5, t = 0.1, p = 0.889) was observed. Joinpoint regression presented evidence of further changes after 2020 with an APC of 230.2 for the last segment (Figure 1C).

## 4. Discussion

The present study reports on the increased demand for CPC observed in the last 10 years at the ED of one of the largest Italian pediatric hospitals. A growing demand for psychiatric care has been reported during the last decade in youth, and an increase in urgent psychiatric presentations for the most serious psychopathology related to suicidal behavior and intent has been registered after the COVID-19 pandemic. This report provides an important contribution to the epidemiological data collection aimed at improving the monitoring, management, and prevention of suicidal behaviors in juveniles, as suggested by the WHO (37).

By analyzing 8,397 consecutive CPCs in ED over more than a decade, we found a highly significant 11-time increase in the number of CPCs ranging from 155 in 2011 to 1,824 in 2021. This corresponds also to a proportional increase of psychiatric indications among all pediatric ED access indications. CPCs corresponded to 0.33% of the total ED access in 2011 vs. 4.15% of the total annual ED access in 2021 (*p* < 0.001, Table 1). CPCs for SA increased significantly by 33 times from 6 in 2011 CPC to 200 in 2021 (3.5% vs. 11% of CPC, *p* < 0.001), and CPCs for SI increased by 32 times from 4 in 2011 CPC to 127 in 2021 (2.6% vs. 7.0% of CPC, *p* < 0.001).

These findings, in line with other US and European epidemiological studies (15, 16, 19), seem to suggest that suicidal behavior and ideation have been a growing phenomenon among Italian adolescents in the last decade. This might be related both to a well-documented general trend of increased incidence of major affective problems among juveniles in high-income countries in the last decades (38).

In the context of the growing demand for psychiatric care for suicidal events in the last decade (12–16, 19), we tried to estimate a possible effect of the COVID-19 pandemic on children and adolescents' mental health by comparing access to the ED for psychiatric reasons in the period before vs. following COVID-19 pandemic outbreak. The total number and the monthly rates of CPCs, CPCs for SI, and CPCs for SA increased significantly from the pre-pandemic period to the pandemic period. We performed further analyses to determine whether the observed excessive increase registered after the pandemic stands out significantly from the previous secular increasing trend with a surplus due to the pandemic.

A joinpoint regression analysis was performed to demonstrate, using a permutation test, whether a model with one or more inflection points in the time trend for each variable significantly fitted to our observations more than a model with a single, constant increasing trend over time. The number of CPCs in ED increased significantly and stably during the study period from 2011 to 2021 with an APC of 21.7, while the total number of ED consultations for any reason increased slightly from 2011 to 2018 (APC of 2.3) and decreased thereafter and during the pandemic (APC of −10.7). Evaluations for SI increased the entire time of the study, with a higher APC (102.8) before 2016 and a milder continuous increase thereafter (APC of 16.3). Evaluations for SA, on the other hand, increased progressively before 2016 (APC of 64.1), were approximately stable from 2016 to 2020 (APC of 1.2), and had a remarkable increase after the pandemic when 200 CPCs for SA were performed in 2021 (APC of 230.2). These findings are compatible with a secular trend in children and adolescents' mental health with a superimposed effect of pandemics, which, hence, had a significant impact on suicide attempts in the pediatric population.

The further increase in SA after the pandemic may be interpreted in our opinion as a true worsening of mental health in youth. Not all studies from developed countries reported an increase in suicide-related access to emergency care, including an Italian study that found CPCs in ED significantly decreased during the first 8 weeks after lockdown (23). Ridout et al. (24) reported only a relative increase in suicidal thoughts and behaviors among all ED presentations of children and adolescents in 2020. This finding seems compatible with a decrease in the overall number of ED presentations rather than with a true increase in suicide-related issues. Some authors (39), discussing a reduction in ED accesses for SA in adolescents during the first lockdown in 2020, hypothesized a compensatory rise of this phenomenon in the post-lockdown period. The methods applied in comparing the annual trend over and the time window considered, which covers more than a decade and extends until the end of 2021, suggests that the increase in SA in 2021 is outstanding and more than a compensatory rise. On the other hand, even if a raw increase in the number of CPCs in ED and CPCs for SI has been observed in the post-lockdown period, on an annual scale, these less serious phenomena may be explained by transient reductions and compensatory rebounds, in a broad outline compatible with the secular trend of increase in demand for psychiatric care in the youngers. Our data on SAs describe a worrying deterioration of adolescents' mental health with an unprecedented excess in most severe psychiatric presentations. This finding is compatible with other recent studies on pediatric mental health during the pandemic reporting an increase in ED consultations for SAs after May 2020, in particular among adolescents more than in adults and among female adolescents aged 12–17 years (27). A Canadian study reported an increase in all mental health-related ED visits in adolescents in the second half of 2020 (25), and a French study on ED accesses of adolescents younger than 16 years found SA to increase from autumn 2020 (26). This is also consistent with another recent study on CPCs in ED in the south of Italy where SA, together with NSSI, was roughly doubled during the second pandemic wave in comparison with the first pandemic wave and to a reference period constituted by the 8 months preceding the pandemic (29). A secular increasing trend has been studied before 2020 also regarding urgent CPCs for SI with some studies reporting a general trend toward an increased rate of major depressive disorders with poor general functioning, comorbid anxiety, eating disorders, and prominent suicidal ideation (13, 40, 41). The relative improvement in the magnitude of the secular increase in SI and SA registered in our setting after 2016 may be partly explained by the parallel improvement of psychiatric healthcare, but this last consideration is not clearly interpretable based on the design of this study and may represent a secondary or spurious finding which needs to be clarified in specifically addressed studies.

Our data highlight, moreover, an increase in psychiatric indications among all ED accesses, with the rate of psychiatric ED consultations divided by all ED consultations peaking at 4.15% in 2021. Moreover, in reports from other authors, psychiatric consultations for different self-harming behaviors represented a significant quote among all ED consultations even if ED consultations for minor urgency for any medical reason decreased (22, 23).

Different from other ecological disasters, the COVID-19 pandemic was characterized by unique circumstances, which represent specific vulnerability factors for mental health. Lockdowns and community activity restrictions have reduced the opportunity for direct social interaction and have modified qualitatively peer relationships for adolescents (42). Moreover, academic interruptions might have contributed to uncertain future perceptions (43). School reopening, resumption of performance requirements and confrontation and competition with peers may represent stress factors accounting for the excess of acute psychiatric presentations registered in 2021. In 2021 in Italy academic activities resumed mostly in presence, after an abrupt shift of all didactic activity to remote learning in the spring of 2020.

Our study has the strength of presenting data from an ED with a large volume of pediatric patient accesses covering a broad time period. Furthermore, psychiatric evaluations were performed clinically, and the evaluation of suicidal ideation and attempts has been standardized with the use of the C-SSRS. These strengths, in our opinion, allow us to draw solid conclusions on epidemiological trends of emergent psychiatric care demand in our population during the last 11 years and particularly after the pandemic in 2020.

Our study has, however, considerable limitations. First, we lack more sociodemographic data on our population. In a recent Australian study (28), an excess increase in post-pandemic ED presentations of children and adolescents for SI and NSSI has been found in higher socioeconomic status populations despite lower socioeconomic status is a well-known vulnerability factor for youth mental health before and after COVID-19 in the general population (44), so the impact of socioeconomic status in mental health and access to different adolescent psychiatric settings of care is a factor which needs to be clarified in other post-pandemic studies. Second, we lack a thorough psychopathological assessment of suicidal attempters. This limits the potential to draw more complete psychopathological conclusions on the excess of SA observed. These limitations are worth to be overcome by following studies to identify the most vulnerable populations to better address public health prevention programs. Furthermore, the study design, by a retrospective chart review, does not allow definite inference about causality. These limitations, however, are shared by most of the cited studies on the same topic, and it is difficult to distinguish among specific co-occurring factors (e.g., economic uncertainty, reduced peer relationship, fear of contagion, and lifestyle changes). Any cause for the observed increase in SA is likely multifactorial, and no definitive conclusions can be made on the relative weight of the pandemic and consequent restrictions to social activities on the observed trends. Another possible limitation to mention is that our single-center cohort may limit the generalization of the results. However, our institution is a referring center covering a large area, also admitting patients from surrounding Italian regions where pediatric psychiatric inpatient units are not available, so changes in the trends described reflect changes in a wide sample from the Italian population.

## 5. Conclusion

In accordance with data from other developed countries, we report a significant increase in the last decade of child psychiatry consultations for any reason as well as specifically requested for juveniles presenting to the emergency department with suicidal ideation or suicide attempt. Consultations for suicide attempts increased significantly between 2011 and 2016, were approximately stable from 2016 to 2020, and then had a peak in 2021 with a greater trend of increase compared to the previous year, probably related to the COVID-19 pandemic.

School closure, routine disruption, loneliness, and delayed access to treatment after the pandemic are possible vulnerability factors. This picture describes a psychiatric pandemic in youngsters, which warrants timely and efficient public health responses, with improvements in emergency settings and mental health resources.

## Data availability statement

The raw data supporting the conclusions of this article will be made available by the corresponding author, upon reasonable request.

## Ethics statement

Ethical review and approval was not required for the study on human participants in accordance with the local legislation and institutional requirements. Written informed consent from the participants' legal guardian/next of kin was not required to participate in this study in accordance with the national legislation and the institutional requirements. This study was conducted in compliance with the declaration of Helsinki, Ethics Committee of Bambino Gesù Children's Hospital practice number 3035/2023.

## Author contributions

SV, AR, UR, MA, and GS contributed to conception and design of the study. MT, EA, FC, RA, MI, AI, MM, CG, and GM collected and organized the data. MA and GS performed the statistical analysis. MA wrote the first draft of the manuscript. MT, EA, and FC wrote sections of the manuscript. All authors contributed to manuscript revision, read, and approved the submitted version.
